# Does a 10-valent pneumococcal-*Haemophilus influenzae* protein D conjugate vaccine prevent respiratory exacerbations in children with recurrent protracted bacterial bronchitis, chronic suppurative lung disease and bronchiectasis: protocol for a randomised controlled trial

**DOI:** 10.1186/1745-6215-14-282

**Published:** 2013-09-05

**Authors:** Kerry-Ann F O’Grady, Keith Grimwood, Allan Cripps, Edward K Mulholland, Peter Morris, Paul J Torzillo, Nicholas Wood, Heidi Smith-Vaughan, Amber Revell, Andrew Wilson, Peter Van Asperen, Peter Richmond, Ruth Thornton, Sheree Rablin, Anne B Chang

**Affiliations:** 1 Queensland Children’s Medical Research Institute, Royal Children’s Hospital, The University of Queensland, Brisbane, QLD, Australia; 2 School of Medicine, Griffith University, Gold Coast, QLD, Australia; 3 Menzies School of Health Research, Charles Darwin University, Darwin, NT, Australia; 4 London School of Hygiene & Tropical Medicine, London, UK; 5 Department of Paediatrics, Royal Darwin Hospital, Darwin, NT, Australia; 6 Royal Prince Alfred Hospital, Sydney, NSW, Australia; 7 National Centre for Immunisation Research & Surveillance, University of Sydney, Westmead, NSW, Australia; 8 Department of Respiratory Medicine, Princess Margaret Hospital, Perth, WA, Australia; 9 Department of Respiratory Medicine, The Children’s Hospital at Westmead and Sydney Medical School, University of Sydney, Sydney, NSW, Australia; 10 School of Paediatrics and Child Health, University of Western Australia, Perth, WA, Australia; 11 Telethon Institute for Child Health Research, Centre for Child Health Research, University of Western Australia, Perth, WA, Australia; 12 Queensland Children’s Respiratory Centre, Royal Children’s Hospital, Brisbane, QLD, Australia

**Keywords:** Bronchiectasis, Child, Chronic suppurative lung disease, Non-typeable *Haemophilus influenzae*, Pneumococcal conjugate vaccines, Protracted bacterial bronchitis, Randomised controlled trial, Respiratory exacerbations, *Streptococcus pneumoniae*

## Abstract

**Background:**

Recurrent protracted bacterial bronchitis (PBB), chronic suppurative lung disease (CSLD) and bronchiectasis are characterised by a chronic wet cough and are important causes of childhood respiratory morbidity globally. *Haemophilus influenzae* and *Streptococcus pneumoniae* are the most commonly associated pathogens. As respiratory exacerbations impair quality of life and may be associated with disease progression, we will determine if the novel 10-valent pneumococcal-*Haemophilus influenzae* protein D conjugate vaccine (PHiD-CV) reduces exacerbations in these children.

**Methods:**

A multi-centre, parallel group, double-blind, randomised controlled trial in tertiary paediatric centres from three Australian cities is planned. Two hundred six children aged 18 months to 14 years with recurrent PBB, CSLD or bronchiectasis will be randomised to receive either two doses of PHiD-CV or control meningococcal (ACYW_135_) conjugate vaccine 2 months apart and followed for 12 months after the second vaccine dose. Randomisation will be stratified by site, age (<6 years and ≥6 years) and aetiology (recurrent PBB or CSLD/bronchiectasis). Clinical histories, respiratory status (including spirometry in children aged ≥6 years), nasopharyngeal and saliva swabs, and serum will be collected at baseline and at 2, 3, 8 and 14 months post-enrolment. Local and systemic reactions will be recorded on daily diaries for 7 and 30 days, respectively, following each vaccine dose and serious adverse events monitored throughout the trial. Fortnightly, parental contact will help record respiratory exacerbations. The primary outcome is the incidence of respiratory exacerbations in the 12 months following the second vaccine dose. Secondary outcomes include: nasopharyngeal carriage of *H. influenzae* and *S. pneumoniae* vaccine and vaccine- related serotypes; systemic and mucosal immune responses to *H. influenzae* proteins and *S. pneumoniae* vaccine and vaccine-related serotypes; impact upon lung function in children aged ≥6 years; and vaccine safety.

**Discussion:**

As *H. influenzae* is the most common bacterial pathogen associated with these chronic respiratory diseases in children, a novel pneumococcal conjugate vaccine that also impacts upon *H. influenzae* and helps prevent respiratory exacerbations would assist clinical management with potential short- and long-term health benefits. Our study will be the first to assess vaccine efficacy targeting *H. influenzae* in children with recurrent PBB, CSLD and bronchiectasis.

**Trial registration:**

Australia and New Zealand Clinical Trials Registry (ANZCTR) number: ACTRN12612000034831.

## Background

Cough is the most common symptom reported for new presentations to primary health care services internationally, [1] and in Australia accounts for 6.8% of family medical practitioner consultations [2]. Chronic cough (>4 weeks duration) [3] in children is associated with increased morbidity and often an unrecognised social and economic burden upon the family and hidden costs for the health care system [4]. There are many causes of chronic cough, but amongst children with a persistent wet cough indicating the presence of excessive airway mucus, there is a subset that has a spectrum of chronic pulmonary diseases. These particular children, if left untreated may develop irreversible airway injury, an event that was well recognised in the pre-antibiotic era [5]. These disorders, protracted bacterial bronchitis (PBB), chronic suppurative lung disease (CSLD) and bronchiectasis, have in common impaired mucociliary clearance, bacterial infection and lower airway inflammation, features consistent with the ‘vicious circle hypothesis’ for the pathogenesis of bronchiectasis [6]. PBB is the most common of these entities, occurring predominantly in preschool children [7,8]. It is characterised by an isolated chronic wet cough without an alternative cause, such as cystic fibrosis (CF) or immunodeficiency being present, which resolves promptly with a two to four-week course of antibiotics [9] In contrast, bronchiectasis is defined as abnormal, irreversible bronchial dilatation and is usually diagnosed by chest high-resolution computed tomography (cHRCT) scans [10,11]. Children with bronchiectasis have a chronic wet cough that responds slowly or incompletely to antibiotic therapy. They also have recurrent lower respiratory infections and can develop additional respiratory symptoms and signs [12]. Bronchiectasis is either idiopathic or results from underlying systemic or local pulmonary diseases predisposing to chronic endobronchial infection. Between these two extremes are children with CLSD who have the clinical features of bronchiectasis, but lack the supporting cHRCT evidence [10]. Recurrent (at least four) episodes of PBB may occur [1], and if these no longer respond promptly to antibiotics, underlying CSLD or bronchiectasis should be considered.

There is a direct relationship between lower airway bacterial load and systemic and lower airway inflammation, risk of acute respiratory exacerbations and quality of life measures in adults with bronchiectasis [13]. *Haemophilus influenzae* (usually unencapsulated or non-typeable (NTHi) strains) is consistently the predominant pathogen found in the sputum of adults with bronchiectasis [14] and chronic obstructive pulmonary disease (COPD) [15-17]. It is also the most common bacterial pathogen isolated from the lower airways of children with PBB, CSLD or bronchiectasis, followed less often by *Streptococcus pneumoniae (pneumococcus)* and *Moraxella catarrhalis*[18,19]. Furthermore, acquiring new *H. influenzae* strains can lead to exacerbations in adults with COPD [15,20,21]. Acute exacerbations are also important in bronchiectasis as they are associated with lower quality of life (QoL) scores [22,23] and poorer long-term outcomes. In adults with bronchiectasis, the frequency of exacerbations, increased systemic inflammatory markers and *Pseudomonas aeruginosa* infection are each determinants of an accelerated pulmonary decline [24]. Amongst other factors, an increased mortality risk is associated with the degree of lung function impairment [25]. In children with bronchiectasis, no prospective study exists, but the only significant predictor of pulmonary decline found in one retrospective study was the frequency of hospitalised exacerbations [26].

While pneumococcal polysaccharide and protein conjugate vaccines seem to have had little impact on the incidence of these chronic pulmonary diseases [8,27], interventions targeting *H. influenzae* may be more successful in reducing exacerbations and leading to improved clinical outcomes. Indeed, adult studies provide “proof of concept” that a *H. influenzae* vaccine may be beneficial. A systematic review of six randomised controlled trials (RCT; 440 patients) reported oral monobacterial whole-cell, killed *H. influenzae* vaccine reduced the incidence of “bronchitis” episodes at three months (rate ratio 0.69; 95% confidence interval (CI) 0.41, 1.14) and six months after vaccination (rate ratio 0.82; 95% CI 0.62, 1.09) [28].

Current oral *H. influenzae* vaccines are not licensed and to date have not been tested in children. Although very different to a whole cell oral vaccine whole cell, oral vaccine, the only vaccine available for children at present that may impact upon *H. influenzae* infection is the parenteral 10-valent pneumococcal-*H. influenzae* protein D conjugate vaccine (PHiD-CV; Synflorix®, GlaxoSmithKline Biologicals, Rixensart, Belgium). The protein D (PD) component is an outer membrane lipoprotein, which is antigenically conserved, surface located and present in most *H. influenzae* (encapsulated and NTHi) strains [29]. It is one of three *H. influenzae* proteins (PD, P6 and OMP26) that have been the focus of potential vaccine antigens for both adults and children in recent years [30,31], with PD and P6 showing the most promise. Vaccine-induced anti-PD antibodies have been associated with protective efficacy against *H. influenzae* infection in middle ear and pulmonary clearance in rat disease models [32]. A RCT of an 11-valent prototype for PHiD-CV (Pneumococcal Otitis Efficacy Trial (POET)) where children had tympanocentesis during their first episode of acute otitis media (AOM) found that the vaccine reduced the overall incidence of AOM by 34%, including a 35% reduction in *H. influenzae*-related cases [33].

However, since our trial commenced, data have been published from a trial of PHiD-CV in 780 Dutch children [34], and GlaxoSmithKline has made aggregate data from a large trial of the vaccine in Latin American infants (COMPAS) publicly available [35]. In the Dutch study [34], infants received the vaccine at 2, 3, 4 and 11 to 13 months of age. Although the study found a lack of vaccine efficacy (VE) against *H. influenzae* (principally NTHi) nasopharyngeal colonisation (VE: 0.5%, 95% CI, -21.8%, 18.4%) and acquisition (VE: 10.9%, 95% CI, -31.3%, 38.9%) no disease endpoints were reported. In COMPAS, vaccine was administered at 2, 4, 6 and 12 to 15 months of age and, despite inducing high serum anti-PD antibody levels, no reduction in *H. influenzae* nasopharyngeal carriage was observed at each three-monthly time point following the third dose of vaccine up until the study ceased when children reached two years of age [35]. There was a marginal effect on any clinically confirmed AOM (VE 16.1%, 95% CI −1.1, 30.4), but none observed for *H. influenzae* confirmed AOM (VE 15%, 95% CI −83.8, 60.7).

The data from COMPAS are difficult to interpret given a surprisingly low prevalence of *H. influenzae* nasopharyngeal carriage in all children at each time point (approximately 5%), and that carriage data were only obtained in a subset of children from a single participating centre (n = 2,000). Furthermore, of all clinically confirmed AOM episodes (n = 243), *H. influenzae* was only detected in 26 (10.7%), of which all were confirmed as NTHi. Hence, there is a clear lack of study power to address the clinical efficacy of the vaccine. The implications of these findings [34] and those of the Dutch study for older children, particularly those with chronic respiratory disease, need to be established and more studies are required in different populations with differing *H. influenzae* epidemiology to confirm vaccine efficacy.

Thus, our trial aims to determine whether respiratory exacerbations in children with recurrent PBB, CSLD and bronchiectasis can be reduced by PHiD-CV vaccination.

### Objectives of the study

Our primary objective is to determine the clinical efficacy of PHiD-CV in reducing respiratory exacerbations in children aged 18 months to 14 years with recurrent PBB, CSLD or bronchiectasis.

Our secondary objectives are:

1. To evaluate the impact of a PHiD-CV vaccine on nasopharyngeal carriage and bacterial load of *H. influenzae* and pneumococcal vaccine-type and vaccine-related serotypes at 2 months post-vaccine dose 1 and then at 1, 6 and 12 months following the second vaccine dose in children with recurrent PBB, CSLD or bronchiectasis.

2. To evaluate the systemic and mucosal immune responses to PD and non-vaccine type *H. influenzae* proteins (P4, P6) and PHiD-CV pneumococcal vaccine and vaccine-related serotypes at 2 months post-dose 1, and then at 1, 6 and 12 months following the second vaccine dose in children with recurrent PBB, CSLD or bronchiectasis.

3. To determine the effect of PHiD-CV vaccine on lung function in children with recurrent PBB, CSLD and bronchiectasis.

4. To evaluate the safety of PHiD-CV vaccine and meningococcal (ACYW_135_) conjugate vaccine in children with recurrent PBB, CSLD or bronchiectasis.

Our study tests the primary hypothesis that amongst children aged 18 months to 14 years with PBB, CSLD or bronchiectasis, vaccination with PHiD-CV reduces the incidence of respiratory exacerbations in the 12 months following two doses of vaccine compared to children who received the control (meningococcal ACYW_135_ Sanofi Pasteur, Lyon, France) conjugate vaccine.

## Methods

### Study design

This is a multicentre, parallel-group, double-blind RCT (with concealed allocation) to assess the efficacy of PHiD-CV in reducing respiratory exacerbations in children with recurrent PBB, CSLD and bronchiectasis. The study plan is summarised in the figure.

### Eligibility

*Inclusion criteria* are:

1. Aged 18 months to 14 years inclusive with recurrent PBB, CSLD or bronchiectasis.

2. Receipt of meningococcal C conjugate vaccine at least six months before enrolment;

3. Negative urine pregnancy test if post-menarchal female.

4. Provision of written informed consent from parent/guardian (assent if child aged ≥10 years).

5. Parent/child willing and able to meet the requirements of the protocol.

6. Access to a telephone and not planning to move from the study area in the 14 months post-enrolment.

7. Has experienced two or more respiratory exacerbations in the 18 months prior to study entry.

*Exclusion criteria* are: chronic lung conditions other than co-existent asthma and those under investigation in this study; prior vaccination with PHiD-CV vaccine; received the 23-valent pneumococcal polysaccharide vaccine (Pneumovax-23, Commonwealth Serum Laboratories Biotherapies, Melbourne, Victoria, Australia) within the previous two months; contraindication/known hypersensitivity to PHiD-CV and/or quadrivalent (ACYW_135_) meningococcal conjugate vaccine; immunosuppressive condition or immunodeficiency disorder that may influence responses to vaccines; systemic immunosuppressive therapy; administration of immunoglobulins and/or blood products within 90 days of receiving study vaccine; active participation in a clinical trial of another investigational drug/vaccine or therapy; acute illness at the time of enrolment, and other medical conditions that could increase the risk of serious adverse events (SAEs) from being in the study.

### Recruitment

Eligible children will be identified from specialist respiratory clinics in tertiary paediatric hospitals in Brisbane, Perth and Sydney, Australia. Parents will be approached by study personnel and informed consent/assent obtained.

### Intervention, follow-up and data collection

**Figure 1:**
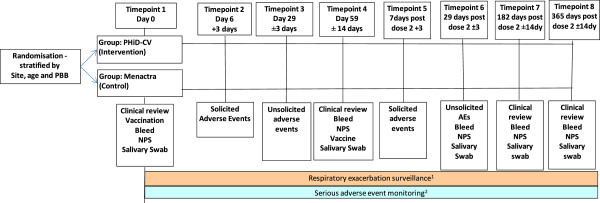
Overall schematic study plan.

Children will be randomised (1:1 allocation) to receive either PHiD-CV or meningococcal (ACYW_135_) conjugate vaccine. Following baseline assessment of their general medical history, respiratory status (including spirometry for children aged ≥6 years), concomitant medication use and vaccination history, children will receive two vaccine doses, 2 months apart, and followed for 12 months post the second vaccine dose. Follow-up includes: three further clinical visits (1, 6 and 12 months post-dose-2); fortnightly telephone interviews with parents to monitor respiratory status; local and systemic adverse event monitoring using parent-completed daily diary cards for 7 and 30 days, respectively, after each vaccine dose; and surveillance for SAEs for the entire study period. All data will be recorded on standardised forms. The primary and secondary outcome measures are collected at the time points specified in Figure 1.

A respiratory exacerbation is defined as an increase in sputum volume or purulence, or three or more days of change in cough (>20% increase in cough score or type (dry to wet)). This definition has been validated, with excellent kappa values between senior clinicians (>0.75) for these symptoms and signs [26]. In addition to active surveillance, parents will be trained in recognising the features of an exacerbation and asked to report any potential episodes that are not captured during the fortnightly follow-up contacts.

### Randomisation, allocation and blinding

An independent biostatistician prepared the randomisation code using a permuted blocking design (block size of four) to maintain group balance. Randomisation was stratified by site, age (less than six years and six or more years), and PBB vs CSLD/bronchiectasis. The age of six years was chosen as at this age spirometry can be performed reliably in children. Treatment allocation is determined by the trial pharmacist at the Royal Children’s Hospital, Brisbane based on the randomisation list provided by the independent statistician. At the time of randomisation, the child’s study specific stratum is confirmed and a request for randomisation based on that stratum is provided to the pharmacist. The trial pharmacist selects the next consecutively numbered opaque sealed envelope from the relevant strata pack, opens the envelope and extracts the randomisation code. This is checked against the randomisation list and a vaccine pack matching the allocation code is assigned to the participant. The allocation sequence is concealed at all times throughout the study from all blinded investigators, study staff and participants.

Since the vaccines differ in colour, their preparation and delivery will be performed by nurses at each participating centre who are independent of the study. Unblinding of individual participants will only occur in emergency situations and ideally after discussion with the Data Safety Monitoring Committee (DSMC). The randomisation code will be unblinded once the database is locked and the data analysed.

### Specimen collection

All children will have nasopharyngeal swabs, saliva and serum collected at enrolment and at 2 months post-vaccine dose 1, and then at 1, 6 and 12 months following the second study vaccine dose (Figure 1). Nasopharyngeal specimens will be obtained with flocked swabs following World Health Organization (WHO) Guidelines [36] and placed in 1.0 ml of skim-milk-tryptone-glucose-glycerol broth. Saliva will be collected using large cotton-tipped swabs, which when soaked are put in a 15 ml tube and placed immediately on ice, while serum will be separated into multiple aliquots. All three specimen types from each child will be stored at local laboratories at −80°C before being transported frozen to the research laboratories.

### Laboratory methods

#### Nasopharyngeal swabs

Batches of swabs will be thawed and 10 μL aliquots cultured overnight on selective media at 37°C in 5% CO_2_. *H. influenzae* and *S. pneumoniae* will be confirmed by standard techniques and routine susceptibility testing performed using the calibrated dichotomous susceptibility disc diffusion method [37]. Diversity of *H. influenzae* isolates will be determined by polymerase chain reaction (PCR)-ribotyping, while the Quellung method (antisera from Statens Serum Institute, Copenhagen, Denmark) will be used to serotype pneumococcal isolates [38].

Real-time quantitative PCR assays will be used to estimate *H. influenzae* and *S. pneumoniae* loads in nasopharyngeal specimens using an in-house multiplex assay. The *H. influenzae* target is *hpd*[39], which also helps differentiate between *H. influenzae* and the commensal *H. haemolyticus,* while the target for *S. pneumoniae* is *lyt*A [40]. Quantitative standards will be generated from the reference strains *S. pneumoniae* (ATCC 49619) and *H. influenzae* (ATCC 19418) obtained from the American Type Culture Collection.

#### Saliva swabs

Saliva antibodies will be measured against purified *H. influenzae* (PD, P4 and P6) and pneumococcal (CbpA, PspA1 + 2 and pneumolysin) proteins and pneumococcal vaccine serotype (1, 4, 5, 6B, 7F, 9V, 14, 18C, 19F, 23F) and serotype-related (6A, 19A) polysaccharides using a multiplex bead assay [41]. Polysaccharide antigens will be conjugated to poly-L-lysine (Sigma Aldrich, Castle Hill, NSW, Australia) using a two-step carbodiimide reaction. All antigens will be covalently bound to carboxylated microspheres (Bio-Rad Laboratories, Gladesville, NSW, Australia) of a specific fluorescent region. Saliva will be diluted in adsorption buffer (phosphate buffered saline (PBS) 0.05% Tween 20 and 2% calf serum; Sigma Aldrich), pneumococcal cell wall polysaccharide (Statens Serum Institute, Copenhagen, Denmark) and serotype 22F polysaccharide (American Type Culture Collection, Manassas, VA, USA) for the polysaccharide assays and straight adsorption buffer for protein assays. Diluted samples will be incubated with antigen-conjugated microspheres at room temperature then washed (PBS 0.05% Tween 20). For detection of IgG or IgA, *R*-phycoerythrin conjugated anti-human IgG or IgA (Jackson ImmunoResearch Laboratories, West Grove, PA, USA), respectively, will be used. The fluorescence in each specific bead region will be measured using the BioPlex® 200 System (Bio-Rad Laboratories). Data will be acquired electronically in real-time and analysed using Bio-plex Manager 5.0 software.

#### Serum samples

Serum antibodies will be measured against purified *H. influenzae* and pneumococcal proteins and pneumococcal polysaccharides outlined above using a multiplex bead assay as described previously [42].

To supplement pneumococcal multiplex bead assays, multiplex opsonophagocytic killing assays will be performed according to the protocol of the WHO Reference Laboratory for pneumococcal serology at the University of Alabama. Only serum from children who have not taken antibiotics within three days of venesection will be tested. Briefly, serially diluted serum samples will be incubated with four pneumococcal serotypes, each with different antibiotic susceptibilities, for 30 minutes at room temperature on an orbital shaker. Samples will be incubated with both rabbit complement and HL-60 cells at 37°C with 5% CO_2_ for 45 minutes. Following incubation, phagocytosis will be stopped by placing microtitre plates on ice for 20 minutes. The reaction mixture will be spotted onto Todd-Hewitt Yeast Agar plates and the mixture left to absorb into the agar for 20 minutes at room temperature. The antibiotic (either optochin, spectinomycin, trimethoprim or streptomycin) containing overlay will be added to each of the plates and incubated at room temperature for 20 minutes. Plates will then be incubated upside down in a candle car for 16 to 18 hours at 37°C. Colonies will be counted by an automatic colony counter and data analysed using the Opsotitre 3 software (University of Alabama, Birmingham, Alabama, USA). Opsonisation indices will be calculated using linear interpolation of the serum dilution killing 50% of bacteria.

### Lung function

Lung function in children aged six or more years will be measured with a standard spirometer using American Thoracic Society criteria at enrolment and 6 and 12 months following the second vaccine dose.

### End points

Participation is completed 12 months (± 14 days) following the second vaccine dose. Other exit points are serious protocol violations and SAEs deemed by the treating clinician or the DSMC to be associated with the study vaccine. Children meeting the exit criteria will continue to be followed until the end of the trial period.

### Outcome measures

#### Primary outcome

The incidence of respiratory exacerbations in the 12 months following the second vaccine dose.

#### Secondary laboratory outcomes

Nasopharyngeal carriage and load of *H. influenzae* and *S. pneumoniae* and *s*ystemic and mucosal immunity to *H. influenzae* proteins (PD, P4, P6) and PHiD-CV pneumococcal vaccine-type and vaccine-related serotypes at 2 months post-dose 1, and then at 1, 6 and 12 months following the second vaccine dose.

#### Secondary lung function outcomes

Spirometry at enrolment and 6 and 12 months after the second vaccine dose.

#### Secondary safety outcomes

Local injection site and systemic reactions in the 7 and 30 days following each vaccine dose, respectively, and SAEs throughout the entire study period.

### Sample size

Prospective data collected in Brisbane children with non-CF bronchiectasis indicate a mean annual incidence of 2.1 (standard deviation 1.046) exacerbations requiring hospital clinic attendance or hospitalisation. PHiD-CV efficacy against exacerbations in children with bronchiectasis is unknown. However, based on the aforementioned pilot data and AOM data from the POET study (30% reduction) [33], our trial is powered to detect a 30% reduction from 2.1 to 1.47 exacerbations in the 12 months following the second vaccine dose. Assuming a Poisson distribution, 93 children per group will provide 90% power (α = 0.05, two-sided) to detect a 30% reduction in exacerbations in the PHiD-CV group and 80% power to detect a 25% reduction. Assuming a 10% loss to follow-up, we will recruit 206 children (103 per group). Differences of less than 20% are unlikely to change clinical practice without additional supporting evidence.

### Analysis

Data will be presented in accordance with the updated CONSORT criteria [43]. Demographic data will be tabulated and expressed as proportions and/or means of the selected characteristics by vaccine group with the corresponding 95% confidence intervals. Differences between groups will be assessed by the normal test for comparisons of means and χ^2^ tests for comparison of proportions.

The primary analysis will assume a Poisson distribution and will compare the incidence of respiratory exacerbations between the two groups. Vaccine efficacy (VE) (%) will be calculated as (1 – Relative Risk (RR))*100 where RR represents the ratio of the incidence of exacerbations in the PHiD-CV group to the incidence in the control group (and presented with its 95% CI). All analyses will be performed on an intention-to-treat basis and will account for baseline stratification.

#### Secondary analyses

The proportion of children with nasopharyngeal carriage of *H. influenzae* and *S. pneumoniae* at each time point will be compared between vaccine groups and presented as RR and 95% CI, adjusted for repeated measures. VE (%) against carriage will be calculated as (1-RR)*100 and presented with its 95% CI. Bacterial load at each time point will be compared.

Serum and mucosal antibody responses to *H. influenzae* proteins, including PD, pneumococcal protein and polysaccharide antigens and opsonophagocytic killing assays: geometric mean titres at each sampling time point will be compared between groups presented as a mean difference (and 95% CI) from linear regression models. In addition, the proportion of children assessed to be seropositive will be compared between groups at each sampling time point using χ^2^ tests for proportions and logistic regression models to adjust for stratification. All differences will be presented with their 95% CIs.

Safety and reactogenicity of vaccination: descriptive analyses will be performed on the number, type and severity of local and systemic reactions and SAEs that occur following vaccination and presented by treatment group. Comparisons between groups will be performed using Student’s *t*-test for continuous variables and χ^2^ tests for proportions.

Sub-group analyses will be performed for all primary and secondary objectives to examine potential differences by study specific strata. Univariate and multivariate analyses will be performed to evaluate variables independently associated with study endpoints and to assess potential confounding factors in the association between vaccination and disease.

### Data safety monitoring committee

A DSMC has been established and has met prior to commencing this study. *A priori* stopping rules include:

#### Efficacy

A Fleming-Harrington-O’Brien [44] stopping boundary will be used at the interim analysis (described below), with a nominal *P*-value required for significance of .001. The final analysis will be referred to a nominal significance level of .0497. An interim analysis of the primary endpoint will be conducted after the first 126 subjects have completed the 12-month follow-up post the second vaccine dose. Sixty-three subjects per group will provide 80% power (α = 0.05, two-sided) to detect a reduction in exacerbations of 30%. At this point, and in discussion with the DSMC, a decision will be made with respect to continuing recruitment based on both the statistical significance and clinical relevance of any effect observed.

#### Toxicity

During the trial, the occurrence of local or systemic reactogenicity may occur at rates that justify additional safety evaluations. If the conditions of any of the stopping rules are satisfied, the data will be assessed fully by the DSMC to determine that it is safe to continue the study. A multi-stage early stopping design approach [45] will be employed to define stopping rules after the occurrence of each observed SAE by comparing the total number of patients included to the maximal number of patients that satisfies maximal acceptable SAE criteria.

### Ethics approval

Human Research Ethics Committees of all the participating institutions have approved the study. It is being conducted under Australia’s Therapeutic Goods Administration Clinical Trial Notification scheme. The University of Queensland is the trial sponsor.

## Discussion

PBB, CSLD and bronchiectasis are important chronic paediatric respiratory illnesses globally. Repeated respiratory exacerbations in those with bronchiectasis are associated with significant long-term morbidity [25,46,47]. There is also historical precedent in the pre-antibiotic era that children with recurrent or persistent wet cough progressed from having normal radiographic appearance to those consistent with bronchiectasis [5]. As *H. influenzae* is the most common bacterial pathogen associated with these diseases in adults and children [18,19], a vaccine that could reduce or eliminate *H. influenzae* may avert exacerbations and reduce antibiotic use. This would be an important adjunct to current clinical management and may lead to short- and long-term health benefits. Our study, therefore, has clinical efficacy as the primary outcome and will be the first to assess the clinical impact of vaccines targeting *H. influenzae* in children with these diseases.

The systemic and mucosal immune responses, particularly antibody functionality, to PHiD-CV in children with recurrent PBB, CSLD and bronchiectasis have not been examined previously. Our study will examine responses to not only PD, but related *H. influenzae* proteins (P4 and P6) that have been considered as vaccine candidates as well as assessing responses to *S. pneumoniae* vaccine and vaccine-related serotype specific antigens*.* A prospective study of serum antibody response to three different *H. influenzae* outer membrane proteins (including PD) during episodes of AOM in otitis-prone and non-otitis-prone children highlights the importance of measuring immune responses in addition to clinical endpoints [48]. It reported diminished IgG responses to these antigens in otitis-prone children compared to non-otitis-prone children at the time of, and following, an AOM episode. Increased susceptibility in otitis-prone children might be related to suboptimal circulating functional T-helper memory and reduced IgG responses to *H. influenzae* or *S. pneumoniae* after colonisation and AOM [49]. These data support ongoing evaluation of responses in children at high-risk of *H. influenzae* infection.

Immunological responses to pneumococcal vaccine antigens are important to evaluate because of recent data suggesting diminished immunogenicity in children who have had either multiple exposures to vaccine antigens [50], or increased density of *S. pneumoniae* nasopharyngeal carriage at the time of vaccination [51,52]. The clinical significance of these observations, however, remains uncertain. A recent study has examined *S. pneumoniae* isolates from bronchoalveolar lavage (BAL) cultures of children with chronic cough thought to be due to PPB [53]. The results suggested significant differences in serotype distribution between 7vPCV vaccinated and unvaccinated children, with immunised children less likely to have vaccine serotypes isolated from BAL cultures, but also more likely to have non-vaccine serotypes than unimmunised children [53]. These findings are consistent with our own observations where pneumococcal conjugate vaccines appear to have had little impact on the burden of lower respiratory tract disease in high-risk Indigenous Australian populations [54], in whom *S. pneumoniae* isolates from nasopharyngeal and BAL cultures are principally non-vaccine serotypes [38,55]. Whether these differences resulted from host responses or population-based changes in circulating serotypes is unknown; however, the study suggests that ongoing surveillance of *S. pneumoniae* in this patient population is required.

It cannot be assumed that existing VE data against *H. influenzae* nasopharyngeal carriage and clinical disease (primarily AOM) in healthy young infants for PHiD-CV, and its 11-valent prototype in POET, can be generalised to preventing acute respiratory exacerbations in the lower airways of older children with PBB, CSLD or bronchiectasis. While the bacterial pathogens invading the middle ear cavities and the lower respiratory tracts of these patients are similar, the bacterial clearance mechanisms and immunity induced following a parenterally administered vaccine may differ between the two anatomical sites. In addition, a recent small study identified significantly reduced natural antibody levels to PD in adult patients with COPD (without exacerbation at time of specimen collection) and secondary immunodeficiency disorders compared to healthy controls, and that natural antibody levels declined with age [56]. Suboptimal circulating functional T-helper memory and reduced IgG responses to *S. pneumoniae* or *H. influenzae* have also been identified in otitis-prone children [49]. These data potentially indicate deficient host responses to *H. influenzae* infection in people with disease and that boosting antibody responses with vaccines may be required. Similar data do not exist for children with chronic lung diseases and, therefore, we have commenced an add-on study to the current protocol to examine this issue.

With respect to differences between nasopharyngeal carriage and disease, the POET study demonstrated a reduction in AOM caused by *H. influenzae*, but not nasopharygeal carriage by this organism in healthy children up to two years of age [33]. However, that study is not comparable to ours given the different population and primary endpoints and, in addition, the vaccine used possessed a higher content of PD than the currently licensed 10-valent PHiD-CV. Furthermore, there remains the possibility of different strains and virulence factors operating between colonisation and disease states [34]. Data comparing these factors between nasopharyngeal and lower airway *H. influenzae* isolates in children with non-CF bronchiectasis or other chronic lung diseases are virtually non-existent. A small study conducted in Australian Indigenous children [38], a population with extensive *H. influenzae* acquisition very early in life [57] and excessive rates of recurrent upper and lower respiratory tract infections [58], found a high density and diversity of respiratory bacteria. It also found nasopharyngeal and BAL strain concordance between the upper and lower airways in 45 children with HRCT confirmed bronchiectasis. However, this study was not conducted at the time of an acute exacerbation and 27 (60%) had received antibiotics within two weeks of specimen collection [38].

Currently, data on PHiD-CV VE against *H. influenzae* carriage and disease in older children are limited to the results from a single study involving healthy four-year-olds [59]. This was a follow-up study of an earlier trial [60] of the PHiD-CV in Czech children conducted when they were 31 to 44 months of age. Persistence of NTHi nasopharyngeal carriage was a secondary objective. Thirty percent of the original cohort (n = 686) did not participate in the follow-up study. Children primed previously with PHiD-CV received a fifth dose at 40 to 48 months of age, the unprimed group received two doses of PHiD-CV two months apart. Post-booster nasopharyngeal carriage data at 24 to 27 months of age from the first study suggested a VE against NTHi carriage of 51.2% (95% CI 11.7, 73.9). However, carriage increased in vaccinees (from 8.5% to 33.5%) and controls (17.3% to 38.9%) at each time point thereafter and, at ages of 31 to 44 months and 40 to 48 months, there was no difference between the two groups [59]. Carriage data beyond those time points have not yet been published.

In addition, the long-term impact of the vaccine on carriage of other microbes is similarly limited. In the same extension study of Czech children, there was no difference between groups in *Staphylococcus aureus* and *M. catarrhalis* carriage over the same time period [59]. This provides an early, albeit insufficient, indication that the PHiD-CV has not altered the dynamics of carriage of these two organisms in healthy young children over time. Whether the same findings occur in children with chronic lung diseases is currently unknown, although we have the capacity to examine these effects in additional laboratory studies outside of the current protocol. Nonetheless, replacement of vaccine type pneumococci by non-vaccine type serotypes has been observed commonly in experimental and non-experimental studies of other pneumococcal conjugate vaccines and in some studies increased carriage of *S. aureus* and *H. influenzae* has also been reported [61,62].

The two study vaccines have well established safety profiles in young children. However, as they are being administered outside of licensed indications in Australia, and to a population of children with existing morbidities, we will undertake a complete safety evaluation during the trial. Our safety endpoints are consistent with those used in trials of both vaccines and SAE monitoring is consistent with Good Clinical Practice criteria [63].

In summary, our RCT will be the first to examine the impact of a vaccine targeting *H. influenzae* in children with recurrent PBB, CSLD and bronchiectasis. The best evidence to answer questions about strain concordance, acquisition of new strains and subsequent infection in the lower airways, and changes in the microbiological profile of acute exacerbations in children with chronic lung diseases following vaccination with PHiD-CV, are best obtained if BALs are performed at baseline, at each study time point and during acute exacerbations. This is ethically unacceptable and, hence, our choice of a clinical endpoint as the primary objective follows the concept of vaccine probe studies as markers of disease burden [64], as demonstrated previously in pneumococcal conjugate and Hib vaccine trials. Clinical efficacy will be evaluated in the context of detailed nasopharyngeal carriage, immunogenicity, lung function and safety data to provide a detailed assessment of whether vaccines may reduce exacerbations and provide an opportunity to improve long-term health outcomes. The study will also be an important contribution to the literature on the effects of conjugate vaccines on the nasopharyngeal microbiology of children with recurrent PBB, CSLD and bronchiectasis.

## Trial status

Recruitment began in January 2013.

## Abbreviations

7vPCV: 7-valent pneumococcal conjugate vaccine; AOM: Acute otitis media; BAL: Bronchoalveolar lavage; CF: Cystic fibrosis; cHRCT: Chest high-resolution computed tomography; CI: Confidence intervals; COPD: Chronic obstructive pulmonary disease; CSLD: Chronic suppurative lung disease; DSMC: Data Safety Monitoring Committee; NTHi: Non-typeable *Haemophilus influenzae*; PBB: Protracted bacterial bronchitis; PBS: Phosphated buffered saline; PCR: Polymerase chain reaction; PD: Protein D; PHiD-CV: Pneumococcal-*Haemophilus influenzae* protein D conjugate vaccine; POET study: Pneumococcal Otitis Efficacy Trial; QoL: Quality of life; RCT: Randomised controlled trial; RR: Relative risk; SAE: Serious adverse event; VE: Vaccine efficacy; WHO: World Health Organization.

## Competing interests

ABC has received Institutional funding from GlaxoSmithKline (GSK) for investigator-led clinical studies in NTHi infections. AWC has participated on Advisory Boards for pneumococcal conjugate and NTHi vaccines for GSK. EKM has participated in Advisory Boards for pneumococcal conjugate vaccines for GSK.

HSV has received funding from GSK to attend scientific meetings. KG has participated in Advisory Boards for pneumococcal conjugate vaccines for GSK. NW has participated in investigator initiated industry supported vaccine studies in the last two years. These have been supported financially by GSK, Aventis Pasteur and Commonwealth Serum Laboratories. He has participated in one GSK- sponsored pertussis meeting in 2011. PM is a member of pneumococcal immunisation and chronic suppurative otitis media Advisory Boards for GSK. RBT has received travel funding from GSK. PR has received funding from GSK for investigator-led epidemiological studies in otitis media and has received travel support from GSK, Wyeth and other vaccine companies to present scientific data and chair workshops. SR, AW, AR, KOG, PJT and PvA have no conflicts of interest to declare.

## Authors’ contributions

KOG conceived the study, devised the study protocol and oversees study implementation nationally and was the primary author of the manuscript. KG made substantial contributions to study conception, grant application, protocol development and implementation and helped draft the manuscript. AWC is responsible for the design and interpretation of the immunogenicity components of the study. EKM, PM and PJT contributed to the grant application and design of the study protocol. NW contributed to the grant application, design of the study protocol and oversees implementation of the study at the Sydney site. HSV contributed to the grant application, study protocol and is responsible for the microbiological components of the study. AR contributed to the grant application, design of the study protocol and assists with study implementation at the Brisbane site. AW contributed to the study protocol and oversees implementation of the study at the Perth site. PR plays a major role in the immunological components of the study. RT is responsible for the immunological assays and interpretation of immunological data. SR is the National Study Coordinator with major input into data instruments, standard operating procedures and GCP compliance. PvA contributed to the grant, study protocol and implementation of the study at the Sydney site. ABC played a major role in study conception, grant application, protocol development and implementation and helped draft the manuscript All authors read and approved the final manuscript.
